# Integrated multi-omics identify key signalling pathways for notochord lumenogenesis in ascidian *Ciona savignyi*

**DOI:** 10.1098/rsob.240402

**Published:** 2025-04-09

**Authors:** Jin Zhang, Zicheng Tan, Qishu Qin, Hongzhe Peng, Wenjie Shi, Haiyan Yu, Bo Dong

**Affiliations:** ^1^ Fang Zongxi Center for Marine EvoDevo, MoE Key Laboratory of Marine Genetics and Breeding, College of Marine Life Sciences, Ocean University of China, Qingdao 266003, People's Republic of China; ^2^ Laboratory for Marine Biology and Biotechnology, Qingdao National Laboratory for Marine Science and Technology, Qingdao 266237, People's Republic of China; ^3^ Institute of Evolution & Marine Biodiversity, Ocean University of China, Qingdao 266003, People's Republic of China

**Keywords:** *Ciona*, notochord, lumenogenesis, Ras-Rap1-MAPK signaling axis, tissue specific transcriptome

## Abstract

Lumen formation and inflation are crucial for tubular organ morphogenesis and function. However, the key signalling pathways for lumenogenesis regulation were not well identified. Here, we performed tissue-specific transcriptomic sequencing for the isolated *Ciona* notochord tissue, in which 10 551 genes in total were identified. To investigate crucial signalling pathways in regulating lumenogenesis, KEGG was performed and the results showed that the Rap1 signalling pathway, vascular endothelial growth factor signalling pathway, mitogen activated protein kinase (MAPK) signalling pathway (plant) and Ras signalling pathway might play important roles in lumenogenesis. Moreover, correlation analysis with proteomic data and comparison analysis of single-cell transcriptomic data were further utilized to identify the potential critical roles of the Rap1 signalling pathway and Ras signalling pathway in lumenogenesis. To verify their functions in lumenogenesis, the Ras/calcium-Rap1-MAPK signalling axis was blocked, and the results showed that the notochord lumenogenesis failed. Meanwhile, we identified that CDC42 was a potential downstream target factor of the Ras-Rap1-MAPK signalling axis, playing crucial functions in notochord lumenogenesis. Overall, we systematically revealed the key regulatory signalling pathways for notochord lumen formation and verified a lumenogenesis-related signalling axis, providing a foundational data resource for exploring the mechanisms of lumenogenesis.

**Figure:**
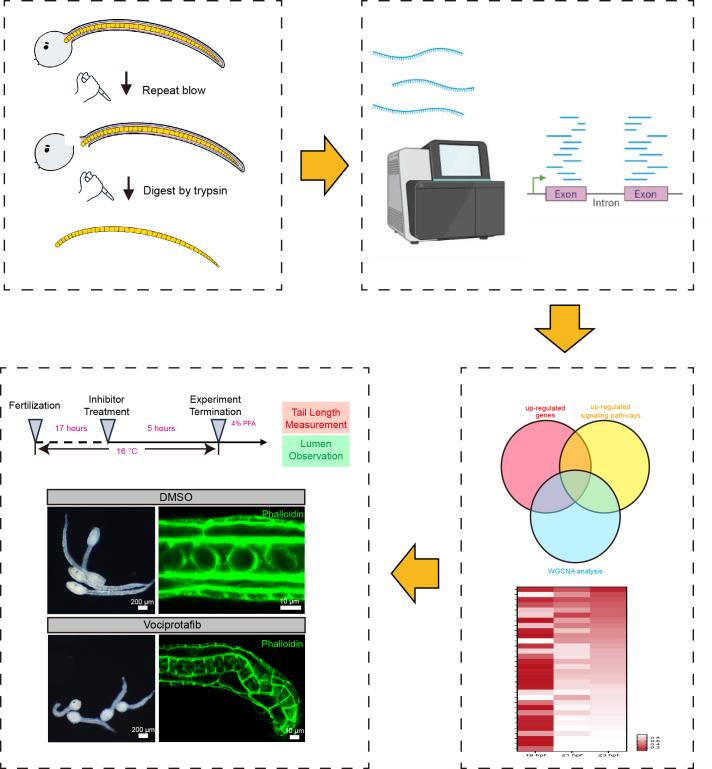

## 1. Introduction

The biological lumen is the basic structural unit that constitutes organs, playing crucial roles in structure support and material transport [1,2]. Many biological processes (BPs) are involved in lumenogenesis, such as the establishment of cell polarity, cell reshaping, vesicle transportation and extracellular matrix (ECM) secretion [2–6]. However, the molecular mechanism and the regulatory signalling pathway controlling lumenogenesis have not been fully identified.

*Ciona*, as a species of chordate, generates a notochord with a typical extracellular lumen at the embryonic and larval stages [4]. The cellular processes of notochord lumenogenesis can be divided into four stages. (i) The first is driven by the actomyosin contractile ring, notochord cells extend along the anterior–posterior axis and narrow along the dorsoventral axis [7]. At the same time, the notochord cells secrete ECM components to form notochord sheath that covers the entire basal surface [8]; (ii) the lumen was initially formed between adjacent notochord cells [9–11]; (iii) the lumen expands driven by luminal fluid secretion. The ECM secreted in luminal fluid is considered an important regulator of lumen expansion [2,8,9,12,13]; and (iv) notochord cells transform to flat endothelial-like cells via bidirectional migration towards both anterior and posterior along the inner surface of the notochord sheath, leading to the connection of isolated lumen [7].

To clarify the key BPs and regulatory molecules for *Ciona* notochord lumenogenesis, omics data have been obtained from *Ciona* notochord tissue. For example, a comprehensive *Ciona* notochord transcriptome has been applied to identify the enriched genes expressed in notochord cells [14,15] and screen the orthologues of *Ciona* notochord genes in vertebrate to explore the notochord evolution in chordates [15]. Recently, the notochord cells were separated through the enzymatic method and were used to perform proteomic sequencing [16]. Based on the proteomic data of *Ciona* notochord tissue, several key BPs during lumenogenesis were identified [16].

In this study, we first isolated notochord tissue from *Ciona savignyi* embryos at 19 (before lumenogenesis), 21 and 23 (during lumenogenesis) hours post fertilization (hpf), using both physical disruption and enzymatic hydrolysis methods. Based on SMART-seq2 technology, we generated transcriptomic data for the notochord tissue of these three stages. We then predicted key BPs and identified primary regulatory signalling pathways during lumenogenesis. We further screened the essential signalling pathways for lumenogenesis by combining the transcriptomic results with proteomic data and the single-cell transcriptomic data of notochord cell clusters in *Styela clava* larvae; one ascidian does not form lumen in notochord tissue [16]. Furthermore, we verified the functions of the identified signalling axis and its potential downstream target protein for lumenogenesis. A signalling axis in regulating *C. savignyi* lumenogenesis was identified, providing a new dataset for research into the mechanism of lumenogenesis.

## 2. Material and methods

### 2.1. Experimental animal and sample preparation

Adult *Ciona* embryos were collected from the coast of Qingdao (Shandong, China) and were stored in seawater tanks in the laboratory. Eggs and sperm from adult animals were dissected and fertilized in seawater. After fertilization, the embryos were dechorionated and cultured in an incubator at 16°C.

*Ciona* tailbud embryos were transferred to a pre-cooled 90 mm dish, coated with 0.2% fetal bovine serum (BSA, Sigma-Aldrich). The embryo’s head was blown off repeatedly with the tip of a pipette. The headless *Ciona* embryos were transferred to a new 90 mm coated dish, and trypsin (Sigma-Aldrich) was added to the dish at a final concentration of 0.8%. Trypsin was dissolved in artificial seawater without Ca^2+^ and Mg^2+^ (Ca^2+^-free ASW, 10 mM KCl, 40 mM MgCl_2_, 16 mM MgSO_4_, 435 mM NaCl, 2.5 mM NaHCO_3_, 7 mM Tris base, 13 mM Tris-HCl) according to previous work [17]. The artificial seawater in the dish was continuously blown with a pipette under a microscope to digest the additional tissues in tailbud embryos. Notochord tissue was collected into a pre-cooled 1.5 ml tube and centrifuged to the bottom to remove the seawater.

### 2.2. RNA extraction and micro-transcriptomic sequencing

Total RNA sample of *C. savignyi* notochord tissue was extracted through the chloroform method. Before performing the transcriptome sequencing, we checked the quality of total RNA through Qubit (concentration of total RNA), Nanodrop (OD value of total RNA) and Agilent 2100 Bioanalyzer (integrity of total RNA). High-throughput sequencing was performed using the Illumina Nova platform in Tianhao Biology Laboratory (Shanghai, China) to obtain FastQ data. Quality assessment of the original sequencing data was performed using FastQC software. The transcriptome data were annotated according to the genomic annotation information of *C. savignyi* from our laboratory [18].

### 2.3. Bioinformatics analysis methods

Deseq2 software was used to analyse differential expression genes between experimental and control groups in pairwise comparisons [19]. Performing differential gene expression analysis on transcriptome data from any two stages, including 19 and 21 hpf, 19 and 23 hpf, 21 and 23 hpf. Differential expression genes are defined as those with *p*-values <0.05 and | log2(fold change) |> 1. Genes with log2(fold change) > 1 are designated as upregulated; genes with log2(fold change) < −1 are designated as downregulated. Genes that do not meet these criteria are labelled as Not DEG (not differentially expressed genes (DEGs)). Gene ontology (GO) and KEGG enrichment for differential genes were analysed according to the background files annotated through the eggnog-mapper (http://eggnog-mapper.embl.de) online platform [20] and visualized using the online platform OmicShare (https://www.omicshare.com/tools/Home/Soft/enrich_circle) [18] and the online tool Hiplot (https://hiplot.com.cn/) [21]. Venn analyses were performed using the jvenn platform (https://jvenn.toulouse.inrae.fr/app/example.html) [22]. Weighted graph co-expression network analysis (WGCNA) was performed using the WGCNA R package on R Studio [23].

### 2.4. Inhibitors treatment experiment

The inhibitors were dissolved in DMSO and added at a final concentration at 17 hpf, and the final concentrations were indicated in the figures. Embryos were cultured at 16°C for about 5 h after adding chemical inhibitors. The treated and control (DMSO treatment) larvae were scored for signs of lumen formation. And the results were the mean of three independent experiments and were performed with the differential analysis. The multiple comparisons by the Dunnett test method were used to analysing the significance between DMSO-treated group and experimental groups (****p < 0.0001).

### 2.5. *In situ* hybridization experiment

*In situ* hybridization was performed at the tail bud stage in *C. savignyi* embryos. Digoxigenin (DIG)-labelled RNA probes were synthesized by *in vitro* transcription with T7 and SP6 RNA polymerase. Embryos were fixed with 4% paraformaldehyde (PFA) in seawater at 4°C overnight and were washed with phosphate-buffered saline containing 0.1% Tween 20 (PBST). The whole-mount *in situ* hybridization was performed by using digoxigenin (DIG)-labelled RNA probes as described previously [24]. The probe primers used in this study are listed in the electronic supplementary material, table S5.

### 2.6. Immunofluorescence staining

Embryos were fixed in 4% PFA at room temperature for 2 h. Permeabilization was then performed by incubating the embryos in PBS containing 0.1% Triton X-100 for a total of 8 h, with three exchanges of solution. After permeabilization, blocking was done using 10% goat serum at room temperature for 1 h. The embryos were incubated overnight at 4°C with the primary antibody (anti-CDC42, 1 : 500). After antibody incubation, the embryos were washed three times in PBS with 0.1% Triton X-100 for a total of 8 h. Next, the embryos were incubated overnight at 4°C with Alexa Fluor 488-conjugated phalloidin (Invitrogen) and the secondary antibody (Goat Anti-Mouse IgG, HRP Conjugate, TransGen, 1 : 2000). After a final set of three washes with PBS containing 0.1% Triton X-100 over 8 h, the embryos were mounted with DAPI for confocal microscopy analysis.

### 2.7. Plasmids construction and electroporation

The full-length coding sequences (CDS) of *cs-cdc42* gene were obtained from the cDNA library of *C. savignyi* through polymerase chain reaction (PCR) with Phanta Max Super-Fidelity DNA Polymerase (Vazyme). CDS fragment purification was performed using the GeneJET gel extraction kit (Thermo Fisher). The CDS fragments were linked to the recombinant pEGFP-N1 vector, replacing the CMV promoter with *cs-brachyury* upstream 3 kb promoter, using the ClonExpress MultiS One Step Cloning Kit (Vazyme) to construct the overexpression plasmid *cs-brachyury(3 kb)*>*cs-cdc42*. The mutant plasmids were generated by introducing mutated bases for overexpression experiments. All the PCR primers are listed in the electronic supplementary material, table S3.

The electroporation method for *Ciona* embryos was performed based on previously reported techniques [25]. Forty micrograms of each plasmid was dissolved in sterile ddH_2_O to a total volume of 80 µl. This plasmid solution was then mixed with 420 µl of electroporation buffer (0.77 M D-mannitol) and was transferred into a 4 mm electroporation cuvette. Subsequently, 300 µl of sea water together with dechorionated *C. savignyi* fertilized eggs were added to the cuvette. Electroporation was performed using a pulse generator, with a voltage setting of 50 V and a capacitance of 1500/2000 µF. After electroporation, the embryos were transferred to a constant-temperature incubator set at 16°C for further incubation.

## 3. Results

### 3.1. Micro-transcriptomic sequencing of the isolated notochord tissues

**Figure 1.**
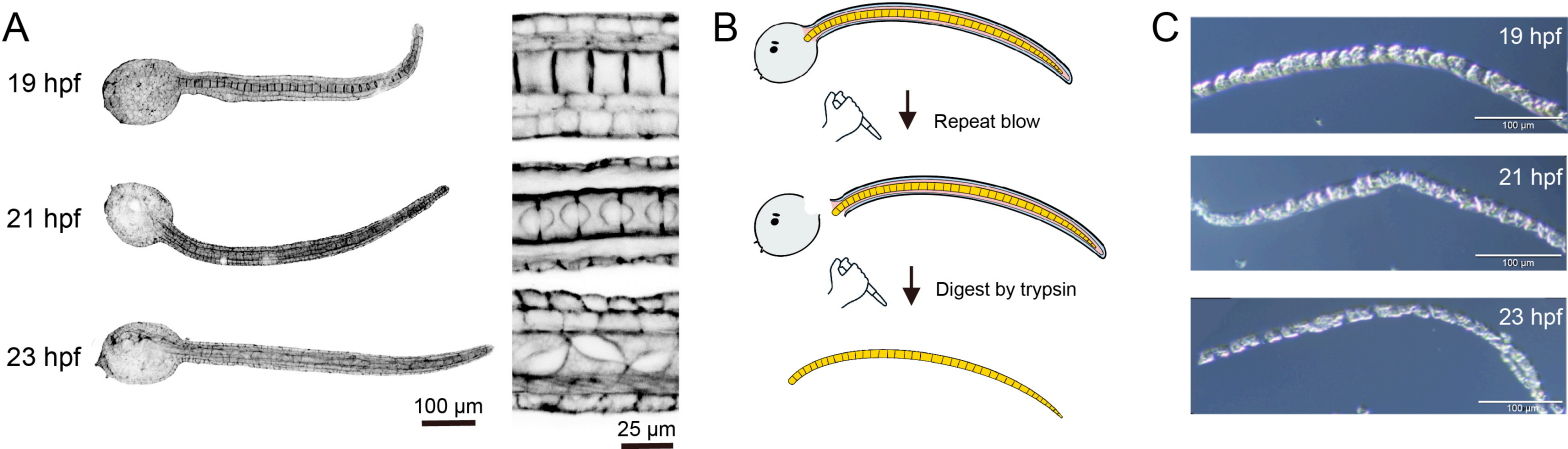
Isolation of notochord tissue from *Ciona savignyi*. (A) Illustration of *C. savignyi* and its notochord tissue at 19, 21 and 23 hours post fertilization (hpf). (B) Workflow of notochord tissue isolated from *C. savignyi* embryos. (C) The isolated notochord tissue from *C. savignyi* tailbud embryos.

In a 16°C environment, 19, 21 and 23 hpf are three key stages for ascidian notochord tubulogenesis. Lumen is not visualizable at 19 hpf, while at 21 hpf, extracellular lumen pockets form at two adjacent notochord cells, and then lumen expands at 23 hpf (figure 1A). To identify the key regulatory factors of notochord lumenogenesis, we collected the notochord tissue at 19, 21 and 23 hpf following the previously published approach [16] (figure 1B). The integrity of notochord tissue was examined by the trypan blue staining method (figure 1C).

SMART-seq2 technology was applied to RNA sequencing of notochord tissue [26]. After constructing a cDNA library by reverse transcription of extracted mRNA, the high-throughput sequencing was performed. Then, the transcriptomic data were annotated based on the reference genome [27]. We set FPKM > 1 as the effective expression of the genes. In total, 10 551 genes were identified to be expressed in *C. savignyi* notochord tissue before and during lumenogenesis (electronic supplementary material, table S1). Wherein, 7452, 10,325 and 10 210 genes correspond to the 19, 21 and 23 hpf embryos and larvae, respectively (electronic supplementary material, table S1).

### 3.2. Dynamics of crucial biological processes during lumenogenesis

**Figure 2.**
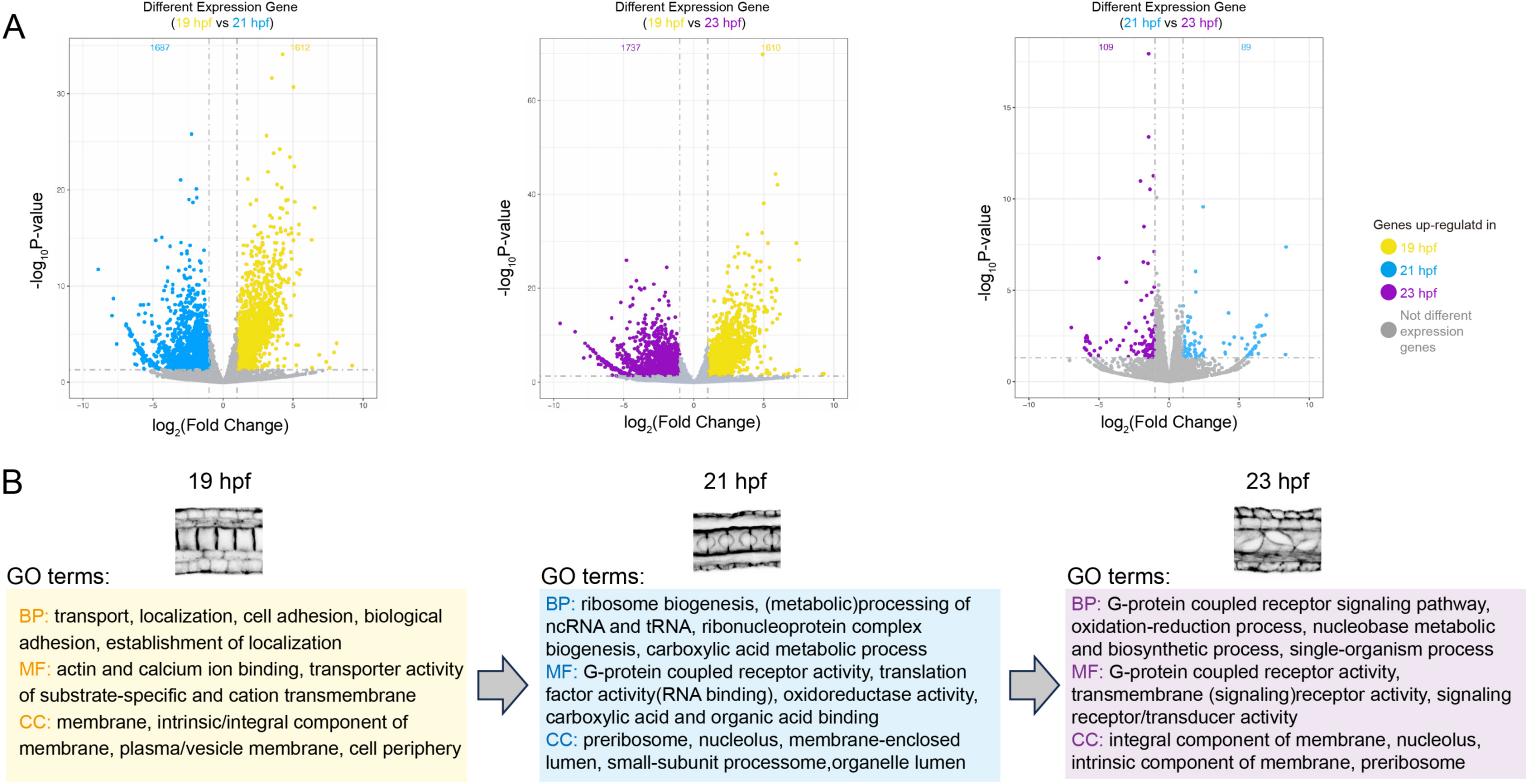
Key biological processes during notochord lumen formation. (A) Different expression genes (DEGs) between 19 and 21 hpf, 21 and 23 hpf, 19 and 23 hpf. DEGs in 19, 21 and 23 hpf are shown in yellow, blue and purple, respectively. Non-DEGs are shown in grey. (B) Summary of top five gene ontology (GO) terms (including biological process, molecular function and cellular compound) at 19, 21 and 23 hpf.

To explore the crucial BPs during lumenogenesis, we exploited the GO analysis to explore the dynamic variation of gene functions during lumenogenesis. The DEGs were enriched. In total, we identified 1787 DEGs in 19 hpf, 1757 DEGs in 21 hpf and 1792 DEGs in 23 hpf (figure 2A). The top 10 GO terms of BP, molecular function (MF) and cellular component (CC) at each stage were summarized and are shown in the electronic supplementary material, figure S1, referring to the *p*-value of GO terms. The gene functions of DEGs at 19 hpf were primarily focused on active material transportation and dynamic alterations (figure 2B). The gene functions of DEGs at 21 hpf were primarily focused on active biogenesis and metabolism (figure 2B). And gene functions of DEGs at 23 hpf were similar to 21 hpf, with a high level of biogenesis and metabolism (figure 2B). Furthermore, we found that the functions of pre-ribosome and nucleolus, the cellular structure and organelle that perform functions in protein synthesis, were more active during lumenogenesis (including 21 and 23 hpf) (figure 2B). These results suggested that the active processes of biogenesis and metabolism contributed to lumenogenesis in *C. savignyi* notochord cells. In addition, compared with 19 hpf, the G-protein-coupled receptor functions were more active at 21 and 23 hpf (figure 2B), implying that the processes related to signal transduction were active during lumenogenesis.

### 3.3. Identification of key signalling pathways for notochord lumenogenesis

**Figure 3.**
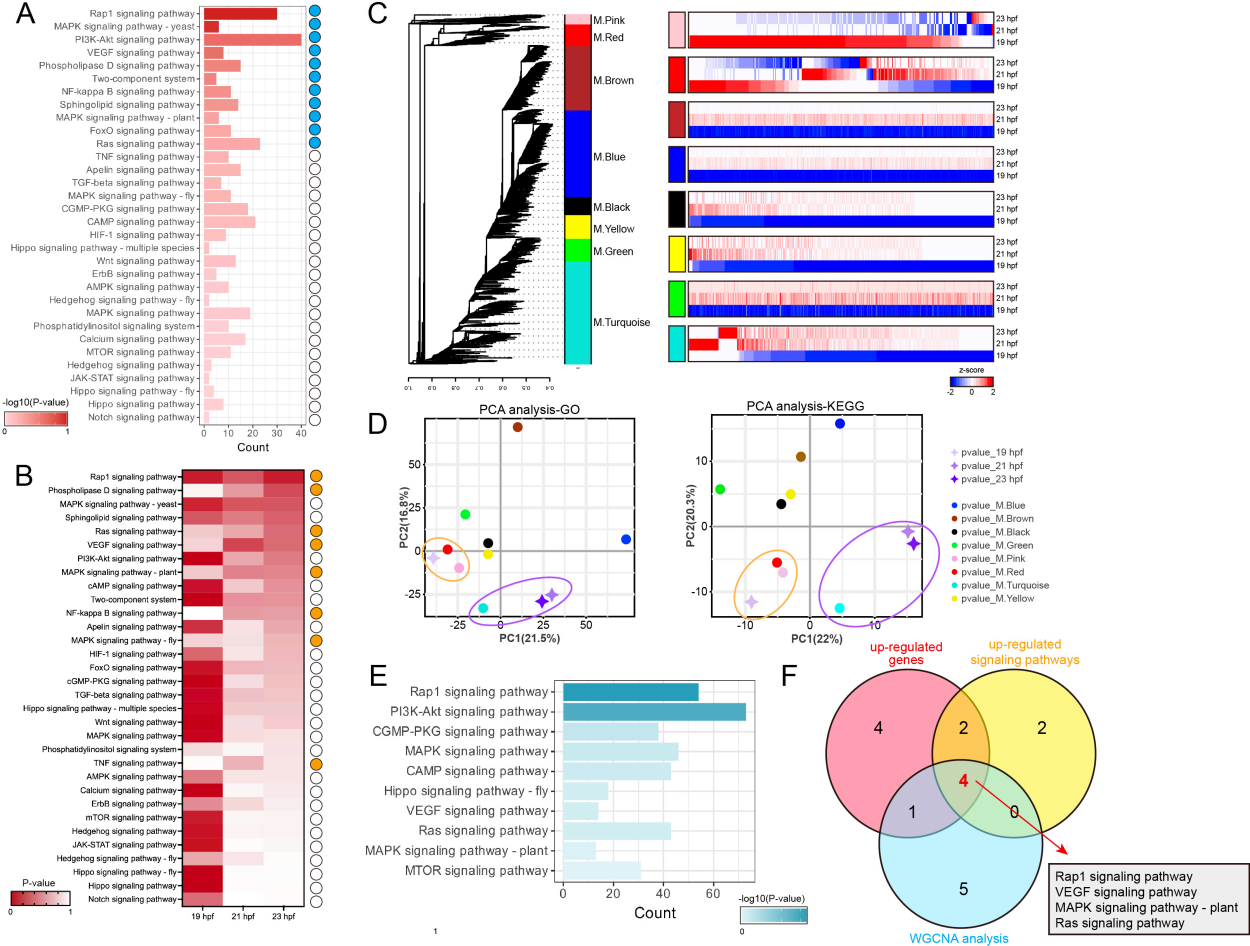
Identification of key signalling pathways for notochord lumenogenesis based on transcriptomic data. (A) KEGG analysis of upregulated genes at 21 and 23 hpf, compared to 19 hpf in *C. savignyi* notochord tissue. We mainly focused on signal transduction-related signalling pathways for further analysis. The deeper red colour represents the lower *p*-value of the signalling pathway, with a tighter relationship with lumenogenesis. The *x*-axis represents the count of upregulated genes in each signalling pathways. The blue spots indicate the signalling pathways with *p*‐value <0.7. (B) The heat map shows the variation of *p*-values of each signalling pathways at 19, 21 and 23 hpf. The deeper red colour represents the lower *p*-value of the signalling pathway, with a tighter relationship with lumenogenesis. The *x*-axis represents different stages. The orange spots indicate the upregulated signalling pathways at 21 and 23 hpf, compared to 19 hpf in *C. savignyi* notochord tissue. (C) Hierarchical clustering dendrogram shows eight co-expression modules of genes that are colour coded according to weighted graph co-expression network analysis (WGCNA). The heat maps show the expression profiles of genes in each co-expression module. The blue colour represents the low expression level, and the red colour represents the high expression level. The intensity of the colour represents log2 and z-score transformed relative expression levels of each gene. (D) Correlation analysis of biological process and signalling pathway between co-expression modules (spot) and developmental stages (four-point star) through principal component analysis (PCA). The orange circle indicates the WGCNA modules with the closest correlation to 19 hpf, and the purple circle indicates the WGCNA modules with closest correlation to 21 and 23 hpf. (E) Top 10 signal transduction-related signalling pathways enriched in turquoise WGCNA module. The deeper turquoise colour represents the lower *p*-value of the signalling pathway, with a tighter relationship with lumenogenesis. The *x*-axis represents the count of turquoise WGCNA module genes in each signalling pathways. (F) Venn diagram shows the critical signal transduction-related signalling pathways both identified in KEGG analysis of upregulated genes (in red circle), upregulated signalling pathways (in yellow circle) and WGCNA analysis (in blue circle). The four critical signalling pathways are shown in the box.

To screen the key signalling pathways involved in signal transduction for lumenogenesis, we performed KEGG analysis for genes that were significantly upregulated after lumen formation. The result revealed that 11 signalling pathways were strongly correlated with lumenogenesis, with *p*‐value < 0.7 (figure 3A). Wherein the Rap1 signalling pathway showed the strongest correlation with lumenogenesis, while PI3k-Akt signalling pathway contributed the most genes involved in lumenogenesis (figure 3A). We further evaluated the correlation between each signalling pathway and lumenogenesis based on the changes in *p*-values at 19, 21 and 23 hpf. We considered that the signalling pathways with lower *p*-values at 21 and 23 hpf, compared with 19 hpf, are more crucial for lumenogenesis. The results showed that eight signalling pathways exhibited reduced *p*-values at 21 and 23 hpf (figure 3B), representing a stronger correlation with lumenogenesis. Wherein, Rap1, phospholipase D, Ras, VEGF, MAPK (plant) and NF-kappa B signalling pathways were both identified through KEGG analysis of DEGs and variation of *p*‐value (figure 3A,B).

To further analyse the transcriptomic data of *C. savignyi* notochord tissue, we performed WGCNA to cluster notochord genes based on their expression profiles and conducted GO and KEGG analysis for each WGCNA modules. The results showed that 10 551 notochord genes were divided into eight modules, in which genes in the brown, blue, black, yellow, green and turquoise modules were upregulated during lumenogenesis; genes in the pink module were downregulated during lumenogenesis and the expression profiles of genes in the red module were significant changed between adjacent stages (figure 3C). Then, we performed principal component analysis (PCA) for the GO and KEGG terms to assess the functional relevance between each stage and the WGCNA modules. The PCA results of GO and KEGG terms were consistent, indicating that the functional annotations of genes in pink and red modules had a stronger correlation with 19 hpf, and the functional annotations of genes in the turquoise module had a stronger correlation with 21 and 23 hpf (figure 3D; electronic supplementary material, tables S2 and S3). The PCA result indicated that genes in the turquoise module might play crucial functions during lumenogenesis. Additionally, we performed the KEGG analysis for genes in the turquoise module and focused on the signalling pathways related to signal transduction (electronic supplementary material, table S3). The results showed that the top 10 signalling pathways related to signal transduction were contained in Rap1, PI3K-Akt, cGMP-PKG, MAPK, cAMP, Hippo (fly), VEGF, Ras, MAPK (plant) and mTOR signalling pathway (figure 3E).

To identify the critical signal transduction-related signalling pathways during lumenogenesis, we take the intersection of the results obtained from the above three analysis approaches, including KEGG analysis of up-regulated genes at 21 and 23 hpf, *p*-value variation of each signalling pathway, and WGCNA analysis. The result suggested that Rap1, VEGF, MAPK (plant) and Ras signalling pathways were identified in all three approaches, implying their crucial roles for *C. savignyi* notochord lumenogenesis (figure 3F).

### 3.4. Identification of key regulatory signalling pathways for lumenogenesis through integrated omics analysis

**Figure 4.**
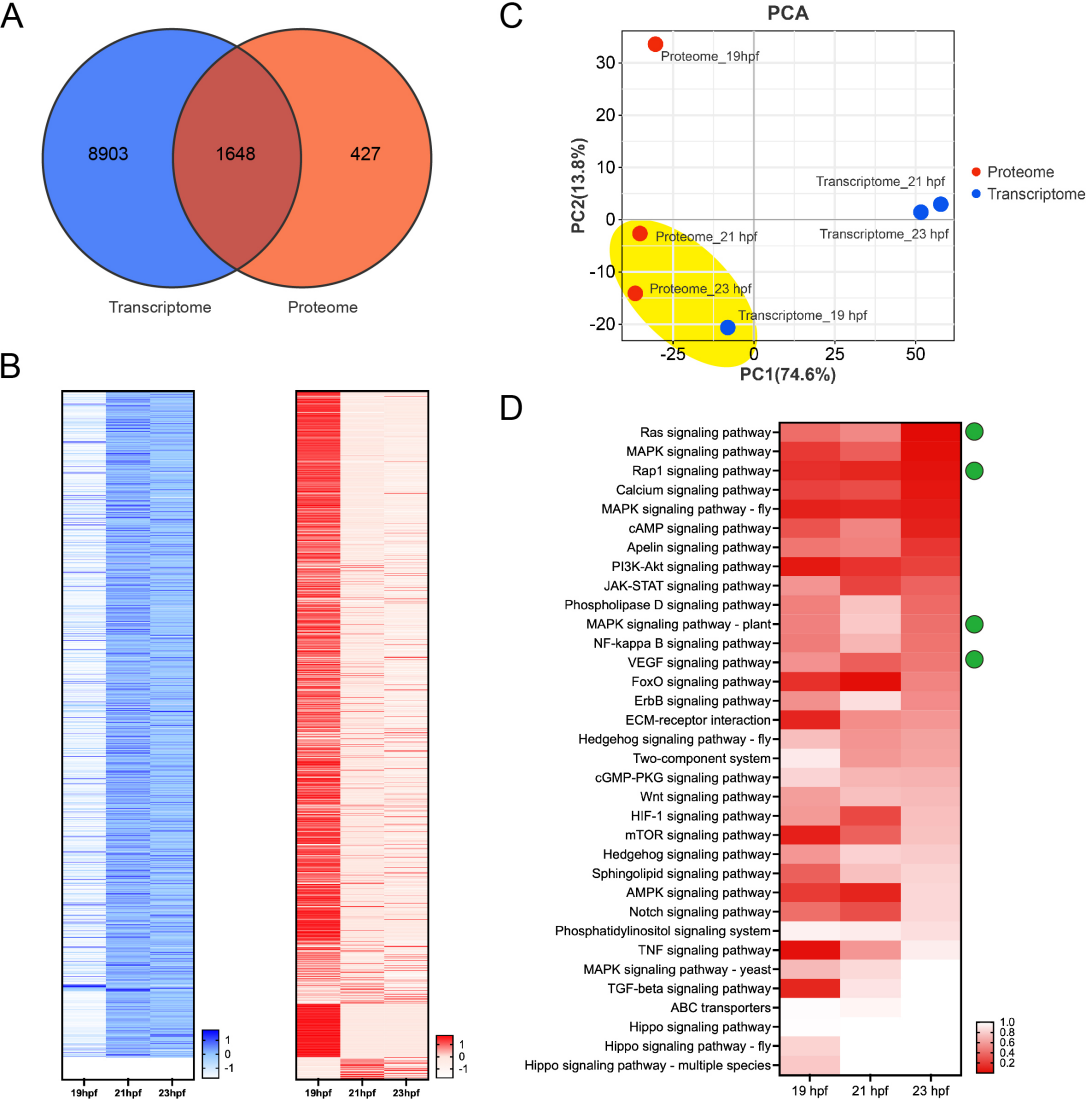
Identification of key signalling pathways for lumenogenesis through comparative analysis with proteomic data. (A) Homology genes and proteins between transcriptomic (in blue) and proteomic (in red) data of *C. savignyi* notochord tissue. (B) Heat maps show the expression profiles of homology genes (according to transcriptomic data, in blue colour) and proteins (according to proteomic data, in red colour). The deeper red or blue colour represents the higher expression level. (C) Correlation analysis of expression profiles during *C. savignyi* lumen formation between transcriptomic and proteomic data through principal component analysis (PCA). (D) The heat map shows the variation of *p*-values of each signal transduction-related signalling pathways at 19, 21 and 23 hpf. The deeper red colour represents the lower *p*-value of the signalling pathway, with a tighter relationship with lumenogenesis. The *x*-axis represents different stages. The green spots indicate the four critical signalling pathways identified by transcriptomic analysis.

The proteomic data of the *C. savignyi* notochord tissue at 19, 21 and 23 hpf were published [16]. To analyse the mRNA–protein correlation and identify key regulatory signalling pathways for lumenogenesis, we performed a correlation analysis between transcriptomic and proteomic data using 10 551 genes in the notochord transcriptome and 2075 proteins in the notochord proteome (electronic supplementary material, tables S1 and S4). Among these data, 1648 genes/proteins were both identified between the transcriptome and proteome (figure 4A). We visualized the expression profiles of these 1648 overlapping genes/proteins in *C. savignyi* notochord tissue at 19, 21 and 23 hpf using a heatmap. The results showed that most of the 1648 overlapped genes were highly transcribed at 21 and 23 hpf based on the transcriptomic data, whereas most of the overlapped proteins were highly translated at 19 hpf (figure 4B). The PCA result also revealed a consistent expression profile between the transcriptome at 19 hpf and the proteome at 21 and 23 hpf (figure 4C), suggesting that protein expression was delayed after mRNA expression.

We further analysed the correlation between lumenogenesis and the four signalling pathways—Rap1, VEGF, MAPK (plant) and Ras—based on their *p*-values at 19, 21 and 23 hpf in the proteome of notochord tissue. The correlation between lumenogenesis and Ras signalling pathway was upregulated from 19 to 23 hpf, and it was the highest at 23 hpf (figure 4D). The correlation between lumenogenesis and Rap1 signalling pathway was constantly high in notochord tissue (figure 4D). The correlation between lumenogenesis and MAPK signalling pathway (plant) was downregulated at 21 hpf, the initial stage of lumen formation (figure 4D). The correlation between lumenogenesis and VEGF signalling pathway was downregulated at 23 hpf, the stage of lumen expansion (figure 4D). Based on the results, the Ras and Rap1 signalling pathways showed higher correlation with lumenogenesis in *C. savignyi* notochord tissue.

**Figure 5.**
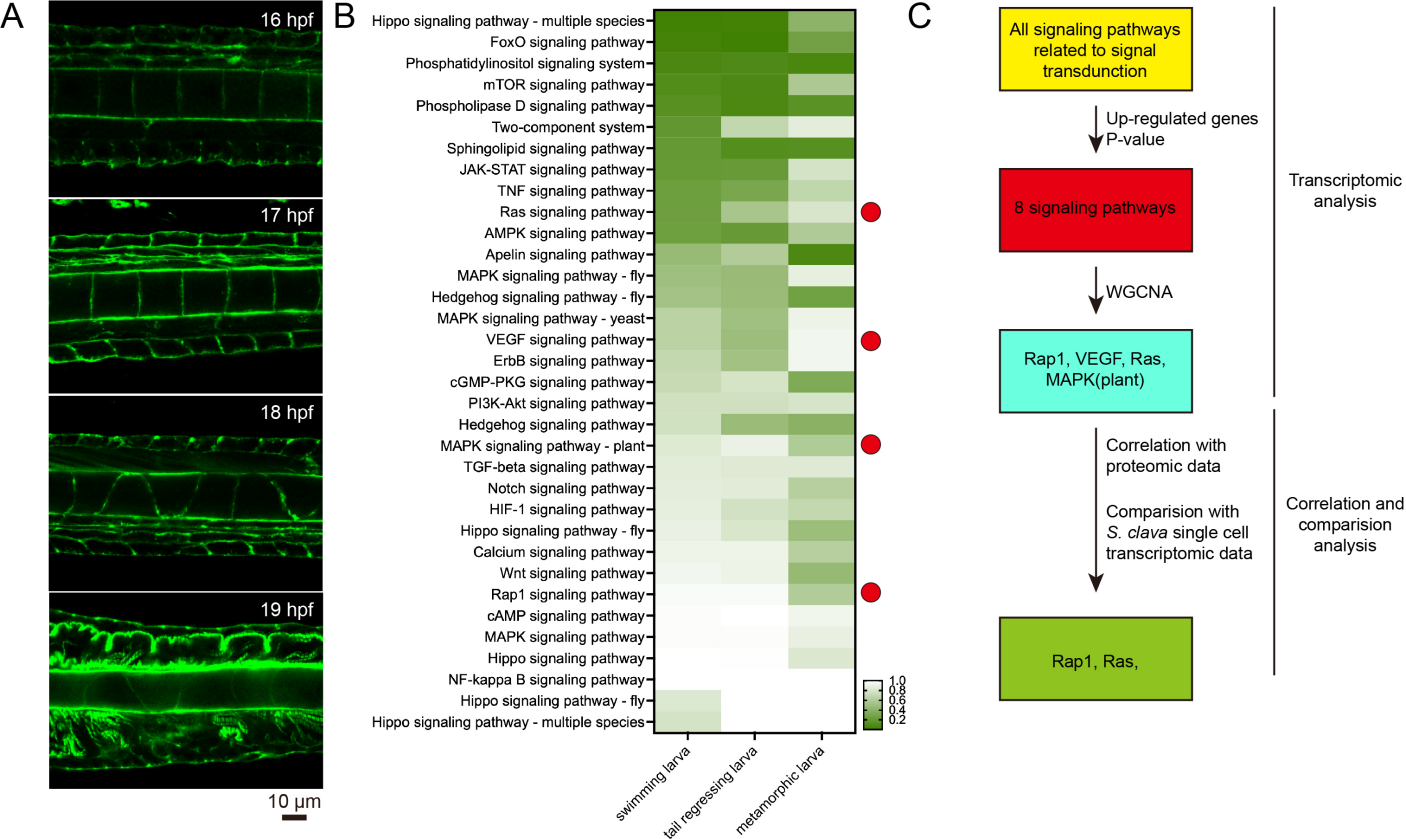
Identification of key signalling pathways for lumenogenesis through species-cross comparison with single-cell transcriptomic data of *Styela clava*. (A) Notochord tissue morphological variation of *S. clava* (one ascidian does not form lumen) in the larval stage. hpf, hours post fertilization. 16−18 hpf, hatched swimming larva. 19 hpf, tail regression larva. (B) The heat map shows the variation of *p*-values of each signal transduction-related signalling pathways in notochord tissue during *S. clava* larval stages. We mainly focused on the notochord cell cluster from single-cell transcriptomic data of *S. clava* for analysis. The deeper green colour represents the lower *p*-value of the signalling pathway. The *x*-axis represents different stages, including swimming larva, tail regressing larva and metamorphic larva. The red spots indicate the four critical signalling pathways identified by transcriptomic analysis. (C) Summary of the screening of signalling pathway related to lumenogenesis.

*Styela clava* is an ascidian species that has notochord tissue without lumen [28]. Phalloidin staining results indicated that no lumen formed at adjacent notochord cells (figure 5A). Accordingly, we hypothesized that crucial signalling pathways for lumenogenesis were likely silenced or downregulated in *S. clava* notochord tissue during the larval stage. Therefore, we performed KEGG analysis for enriched genes in notochord tissue during *S. clava* larval stages, based on the single-cell transcriptomic data of *S. clava* larvae. Among the four signalling pathways identified above, the Ras signalling pathway was downregulated in notochord tissue during *S. clava* larval stages, and the Rap1 signalling pathway was shown to have low enrichment in notochord tissue during *S. clava* larval stages (figure 5B). While the VEGF signalling pathway was upregulated at the tail regression larval stage, the MAPK signalling pathway (plant) was upregulated at the metamorphic larval stage.

Based on the correlation analysis with proteomic data of *C. savignyi* notochord tissue and comparison analysis with single-cell transcriptomic data of *S. clava* notochord tissue, we suggested that the Rap1 and Ras signalling pathway were shown to have high relevance with lumenogenesis in *C. savignyi* notochord tissue (figure 5C).

### 3.5. Ras/calcium-Rap1-MAPK signalling axis plays crucial functions in lumenogenesis

**Figure 6.**
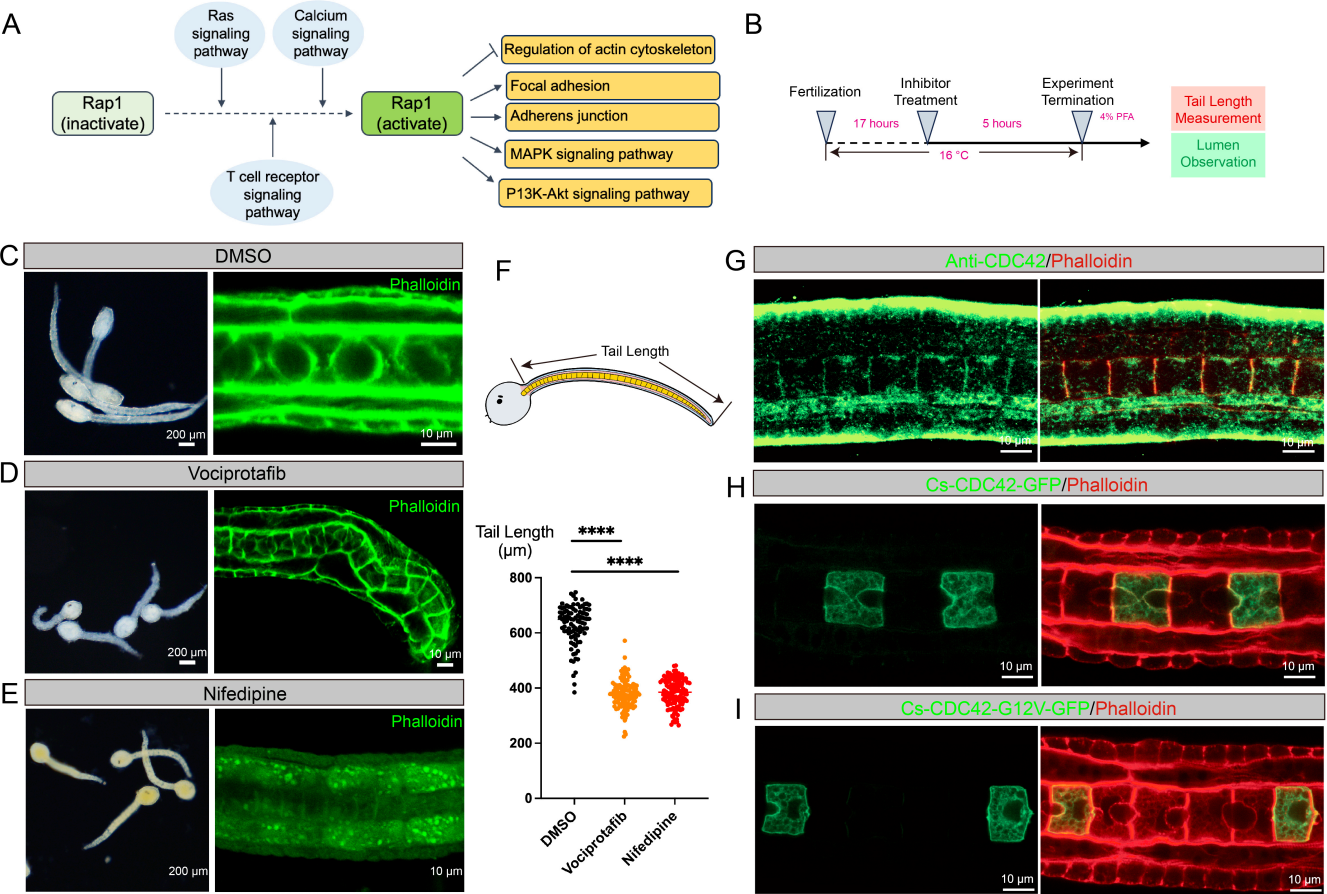
Functional variation of Ras/calcium-Rap1-MAPK signalling axis for *C. savignyi* lumen formation. (A) A regulatory model of Ras-Rap1 signalling pathway. (B) The process of the inhibitor treatment experiment. (C–E) Phenotypes of (C) DMSO (negative control group), (D) vociprotafib (inhibitor of Ras-Rap1-MAPK signalling pathway) and (E) nifedipine (inhibitor of Ca^2+^ signalling pathway) treatment of *C. savignyi* tail bud embryo. The morphologies of the whole embryo are shown on the left, and the morphologies of the notochord tissue are shown on the right. The green signal represents the cytoskeleton. Bars are shown in the lower right corner. (F) Statistics of embryo tail length after inhibitor treatment. The model of the tail length measurement method is shown on the upper part of the figure. The statistical result of *C. savignyi* embryo tail length after inhibitor (including nifedipine, vociprotafib) and DMSO treatment. The results of multiple comparisons (by the Dunnett test) between each inhibitor treatment group and DMSO treatment group were performed, respectively. ****p‐value < 0.001. (G) Immunofluorescence of *C. savignyi* notochord tissue with anti-CDC42. The green signal represented the staining of anti-CDC42, and the red signal represented the staining of phalloidin. Bars are shown in the lower right corner. (H,I) Overexpression of CDC42 proteins (H) and CDC42 proteins with amino acid mutation (I) in *C. savignyi* notochord cells. The promoter of *brachyury* gene was used to drive the target sequence specifically expressed in notochord cells, and the GFP protein was used for staining. The red areas represent the staining of phalloidin. Bars are shown in the lower right corner.

A comprehensive analysis indicated that the Rap1 signalling pathway and Ras signalling pathway played crucial roles in lumenogenesis. The Ras signalling pathway has functions in the activation of Rap1 signalling pathway, which positively regulates the MAPK signalling pathway [29,30] (figure 6A). Our *in situ* hybridization result showed that *Cs-Mras* gene was expressed in *C. savignyi* notochord during lumenogenesis (electronic supplementary material, figure S2A). To investigate the role of the Ras-Rap1-MAPK signalling axis in lumenogenesis, we conducted inhibitor treatment experiments (figure 6B). SHP2 protein is a tyrosine phosphatase and scaffold protein that preferentially binds to and dephosphorylates tyrosyl-phosphorylated RAS proteins, thereby enhancing the activation of RAS protein and activating the Rap1-MAPK signalling axis [30–32]. Vociprotafib is the inhibitor of SHP2 protein [33]. Tailbud embryos at 17 hpf were treated with Vociprotafib (an inhibitor of the Ras-Rap1-MAPK signalling axis) and then cultured in a 16°C developmental environment (figure 6B). Five hours later, we fixed the embryos with 4% PFA and assessed tail length and lumen morphology (figure 6B,F). In the vociprotafib-treated group, the lumens in tailbud embryos were significantly smaller compared to the control group (figure 6C,D; electronic supplementary material, figure S2B), and the notochord was bent at the tail end (figure 6D). These results suggested that the Ras-Rap1-MAPK axis played a regulatory role in lumen expansion and tail elongation (figure 6C,D). Additionally, tailbud embryos treated with vociprotafib resulted in a significant reduction in tail length compared to the control group (figure 6F). Overall, these findings indicated that the Ras-Rap1-MAPK signalling axis was crucial for lumenogenesis in *C. savignyi* notochord.

CDC42, one kind of small GTPase in the Rho protein family, is an important downstream target protein in the Ras-Rap1 signalling axis, and it plays crucial functions in cell adhesion, migration and polarity [29,34]. The immunofluorescence result showed that CDC42 proteins were distributed on both the apical and basal membranes of notochord cells in *C. savignyi* tailbud embryos (figure 6G). On the basal membrane of notochord cells, CDC42 proteins were mainly distributed at the middle region (figure 6G). To investigate the roles of CDC42 proteins during lumenogenesis, we overexpressed the CDC42 proteins with a dominant-negative mutant at a critical amino acid in *C. savignyi* embryo notochord tissue and observed the phenotypes [35,36]. Compared with the control group, the process of lumenogenesis failed, and the lumen formation failed in the mutation group (figure 6H,I), similar to the phenotype with the vociprotafib-treated tailbud embryos (figure 6D). These results suggested that CDC42 proteins, downstream of the Ras-Rap1 signalling axis, play critical roles in lumenogenesis.

In addition to the Ras signalling pathway, the calcium signalling pathway also plays functions in the activation of Rap1-MAPK signalling axis (figure 6A). To confirm its role in lumenogenesis, we performed the inhibitor treatment experiment with nifedipine (calcium signalling pathway inhibitor [37]) following the same protocol (figure 6B). The results showed that notochord lumen formation was impaired (figure 6C,E) in the nifedipine-treated group, and the phalloidin staining signal intensity around the cell membrane decreased (figure 6C,E), indicating that disruption of the calcium signalling pathway led to failure of lumen formation and cytoskeleton disassembly. Besides, the tail length of nifedipine-treated embryos was significantly reduced compared to the control group (figure 6F). Overall, these results suggest that the calcium signalling pathway is also important for lumenogenesis in *C. savignyi* notochord.

## 4. Discussion

The *Ciona* notochord serves as an ideal model for lumenogenesis study. However, the regulatory signalling pathways have not been systematically identified. In this study, we isolated notochord tissue from *C. savignyi* embryos at 19, 21 and 23 hpf, and constructed transcriptomic sequencing using Smart-seq technology. Based on the transcriptomic data, we performed GO analysis to construct the dynamic variation of gene functions before and during lumenogenesis. To identify the crucial signalling pathways related to signal transduction during lumenogenesis, we performed KEGG analysis for significant upregulated genes during lumenogenesis, *p*-value variation of each signalling pathways and WGCNA modules. Our analysis suggested that Rap1, VEGF, MAPK (plant) and Ras signalling pathways showed higher correlation with lumenogenesis in *C. savignyi* notochord. Furthermore, we performed correlation analysis with proteomic data of *C. savignyi* notochord tissue at the same stages, and comparison analysis with single-cell transcriptomic data of *S. clava* notochord cell cluster and suggested that the Rap1 signalling pathway and Ras signalling pathway were critical for notochord lumenogenesis. Eventually, we performed a chemical inhibitor experiment to validate that the Ras/calcium-Rap1-MAPK signalling axis played functions in lumenogenesis. Meanwhile, CDC42, a potential downstream target protein of the Ras/calcium-Rap1-MAPK signalling axis, also plays functions in regulating the lumenogenesis in notochord of *C. savignyi* embryo.

Isolation of notochord-specific cells has been performed using electroporation of the reporter construct Bra>GFP, followed by flow sorting to isolate notochord-enriched GFP^+^ cells from notochord-depleted GFP^−^ cells [15]. This method sorted the notochord cells well from *Ciona* embryos. In this study, to identify the signalling pathways in notochord tubulogenesis, we utilized the approach in this study based on the following considerations: first, the flow sorting method destroyed the notochord cell junctions and the ECM environment, resulting in the changes of gene expression profiles [38]. Second, it might be time-consuming to sort sufficient notochord cells through fluorescence signal, because of the mosaic developmental pattern of *Ciona* embryos [39,40]. Third, due to the weak expression of *brachyury* gene in mesenchyme cells and autofluorescence in *Ciona* embryo, the sorted cells might be mixed with mesenchyme cells [15]. In *Ciona* embryos, the notochord ECM sheath is inherent hardness and maintains the integrity of the notochord and separates it from surrounding muscle and epidermal tissues [38,41,42]. The approach that we developed could collect sufficient and specific notochord tissue with good shape to perform proteomic [16] and transcriptomic sequencing experiments.

A previous study has shown that the vesicles carrying luminal fluid proteins are transported to the central regions of adjacent notochord cells, which was considered as an important mechanism for initiating lumen formation [9,10]. The typical tight junction was also observed between the apical membrane (lumen membrane) and the lateral membrane (cell contact region between notochord cells) to prevent the leakage of luminal fluid [7]. Our results showed that genes related to the substantial transport and cell adhesion activity were enriched at 19 hpf (figure 2B; electronic supplementary material, figure S1A). KEGG analysis showed that signalling pathways related to focal adhesion and tight junctions were significantly enriched during this phase. Considering that protein translation was delayed after mRNA transcription, the processes involved in substantial transport and cell adhesion were highly active during lumenogenesis (electronic supplementary material, figure S1B). According to the GO enrichment analysis, we found that active biosynthesis and metabolism occurred during lumenogenesis (figure 2B; electronic supplementary material, figure S1A), which has a time overlapping with a significant increase of the luminal fluid proteins secretion. These luminal fluid proteins are thought to be ECM proteins [8,16], which aligns with the GO enrichment results for genes in the turquoise module, showing the close functional relevance to the 21 and 23 hpf stages (figure 3D; electronic supplementary material, tables S2 and S3). In summary, the functional enrichment analysis of DEGs was consistent with the observed BPs, supporting the reliability of the analysis.

The result of correlation analysis between the transcriptome and proteome of notochord tissue showed that the transcriptome at 19 hpf exhibited a consistent expression profile with the proteomes at 21 and 23 hpf (figure 4C), suggesting a delay in protein translation relative to mRNA transcription. Therefore, high-expression genes at 19 hpf at the mRNA level probably played functions in lumenogenesis. We further performed the comparison analysis of the signalling pathways between transcriptome and proteome. We found that most of the signalling pathways elevated at 21 or 23 hpf in the proteome will be enriched at 19 or 21 hpf in the transcriptome (electronic supplementary material, figure S3). However, the AMPK, Hedgehog, VEGF, calcium and phosphatidylinositol signalling pathways were elevated at 21 or 23 hpf in the proteome, but those were not enriched at 19 or 21 hpf in the transcriptome, suggesting that these signalling pathways also play roles in lumenogenesis.

In previous research, fibroblast growth factor (FGF)-Ras-MAPK signalling was proved to be required for the induction of notochord, mesenchyme and neural tissues in *C. intestinalis* and *Halocynthia roretz*i [43–47]. Knockdown of *Ci-Mras* with morpholino antisense oligonucleotides in *Ciona* embryos resulted in the abrogation of the *brachyury* expression, one notochord marker gene [14,47]. Concurrently, the exogenous expression of an activated *Ci-Mras* also activates ERK, explaining the conserved regulatory mechanism of RAS between ascidian and mammal [47]. MAPK signalling pathway is required for the formation of various tissues in the ascidian embryogenesis. After treating the ascidian embryo at the 32-cell stage with U0126 (one inhibitor of MEK), the formation of notochord tissue and mesenchyme tissue failed [44]. The results of the *in vitro* cellular experiment also showed that FGF-MAPK signalling has a positive regulatory function on *brachyury* [48,49]. In our research, a Ras/calcium-Rap1-MAPK signalling axis was identified as a key regulatory signal pathway for notochord lumenogenesis. After disrupting the calcium signalling pathway or Ras-Rap1-MAPK signalling axis in *C. savignyi* embryo, the volume of the lumen was decreased, the rate of lumen expansion was slowed down, the extension of the embryonic tail was obstructed, and the notochord tissue was bent (figure 6C–F; electronic supplementary material, figure S2B), suggesting the important function of the Ras-Rap1-MAPK signalling axis in lumen formation. Meanwhile, the *in situ* hybridization results also showed the C*s-Mras* (key gene of Ras signalling pathway) was expressed in the notochord (electronic supplementary material, figure S2A). Thus, we confirmed the core status of the Ras/calcium-Rap1-MAPK signalling axis in lumenogenesis regulation and assumed that *brachyury* gene might be one of the crucial target molecules of Ras/calcium-Rap1-MAPK signalling axis to control the morphology of the lumen and notochord.

The Ras signalling pathway is crucial for regulating energy metabolism through downstream signalling axis, such as Raf-MAPK and PI3K-AKT-mTOR [50–52]. Mice with a germline knock-in expressing H-Ras^G12S^ exhibited impaired hepatic energy homeostasis, along with reduced fatty acid oxidation and altered glucose metabolism [50,53]. Additionally, the Ras-MAPK signalling axis was shown to affect central carbon metabolism through inducing the expression of hypoxia-inducible factor and decreasing the mitochondrial respiration [54,55]. In our study, we suggested that the Ras-Rap1-MAPK signalling axis played crucial roles in lumenogenesis in *C. savignyi* (figure 6C–F, electronic supplementary material, figure S2B). The results of transcriptome analysis also show that the processes of biosynthesis and metabolism are enriched during lumenogenesis. Together, we supposed that the functions of the Ras-Rap1-MAPK signalling axis in lumenogensis were probably related to biosynthesis and metabolism. In mammals, CDC42 protein is one of the Ras-related GTP-binding proteins that regulates the assembly and disassembly of the actin cytoskeleton through responding to the extracellular signals [56]. Our results showed the similar phenotypes between the embryos treated with vociprotafib and those overexpressing CDC42 proteins with a dominant-negative mutant, suggesting that the CDC42 protein is a potential downstream target of the Ras signalling pathway. Based on this, we hypothesized that the CDC42 protein is a conserved downstream target of the Ras signalling pathway in ascidians and mammals.

## 5. Conclusions

In this study, we constructed a notochord tissue-specific transcriptomic map of *C. savignyi* embryos before and during lumenogenesis. Our analysis revealed the key BPs involved in lumenogenesis. By integrating multi-omics data, we identified and verified a critical signalling axis in regulating lumenogenesis: the Ras/calcium-Rap1-MAPK signalling axis. We also found that CDC42 protein might be the potential downstream target protein of the Ras/calcium-Rap1-MAPK signalling axis and that it plays crucial functions in lumenogenesis. This study provides new data to support mechanism research on ascidian lumenogenesis and highlights a new signalling axis that regulates ascidian lumenogenesis.

## Data Availability

The datasets presented in this study can be found in publicly available repositories. The transcriptomic data of *C. savignyi* notochord tissue at 19 hpf, 21 hpf and 23 hpf are deposited in NCBI under the BioProject accession number PRJNA1231721. The single-cell transcriptomic data of *S. clava* at hatched swimming larval stage, tail regressing larval stage and metamorphic larval stage are deposited in NCBI under the BioProject accession number PRJNA1122959. The proteome data of *C. savignyi* notochord tissue at 19 hpf, 21 hpf and 23 hpf are openly available in ProteomeXchange (PXD037089) at http://www.ebi.ac.uk/pride [16]. Supplementary material is available online [57].
